# Hesperidin reduces serum levels of triglyceride after coronary artery bypass graft surgery

**DOI:** 10.1002/fsn3.3639

**Published:** 2023-08-23

**Authors:** Zahra Booyani, Naheed Aryaeian, Negar Omidi, Atie Sadat Khorasanian, Agha Fatemeh Hoseini, Mostafa Nejatian, Shima Jazayeri, Mehrnaz Morvaridi

**Affiliations:** ^1^ Department of Nutrition, School of Public Health Iran University of Medical Sciences Tehran Iran; ^2^ Cardiac Primary Prevention Research Centre, Cardiovascular Disesae Research Institute Tehran University of Medical Sciences Tehran Iran; ^3^ Department of Biostatistics, School of Health Iran University of Medical Sciences Tehran Iran; ^4^ Department of Cardiac Rehabilitation, Tehran Heart Center Tehran University of Medical Sciences Tehran Iran

**Keywords:** coronary artery bypass graft, depression, hesperidin, inflammation, oxidative stress

## Abstract

Hesperidin, as an antioxidant and anti‐inflammatory agent, has beneficial effects on cardiovascular diseases. This study aimed to determine the effects of hesperidin supplementation on inflammation, oxidative stress, and lipid profile in depressed coronary artery bypass graft surgery (CABG) patients. Eighty patients after coronary artery bypass graft surgery participated in this clinical trial and were randomly divided into two groups. The intervention group received 200 mg/d pure hesperidin supplement and the second group received placebo for 12 weeks. Both groups continued their usual diet. Serum concentrations of inflammatory and stress oxidative markers (hs‐CRP, P‐selectin, and ox‐LDL) were measured and compared at baseline and the end of the intervention. The changes in serum levels of triglyceride were significantly different between the two groups (*p* < .05). HDL‐c significantly increased in groups but the differences between the two groups were not statistically significant (*p* > .05). Hesperidin did not affect FBS, other lipid parameters, hs‐CRP, P‐selectin, and OX‐LDL (*p* > .05). SBP and DBP differences were not statistically significant (*p* > .05). After 12 weeks of intervention, hesperidin reduced serum levels of triglyceride in depressed post‐CABG patients.

## Graphical abstract

Hesperidin, as an antioxidant and anti‐inflammatory agent, has beneficial effects on cardiovascular diseases. After 12 weeks of intervention, hesperidin reduced serum levels of triglyceride in depressed post‐CABG patients.

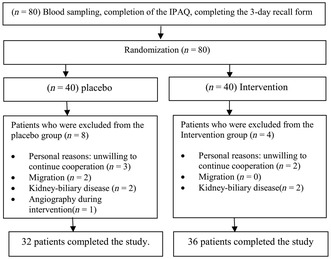

## 1 INTRODUCTION

Cardiovascular diseases are chronic and progressive diseases that cause disability and early death (Ferreira‐Gonzalez, 2014). About 20% of the world's population is affected by the disease (Deyo, 1991). Coronary artery bypass graft surgery (CABG) is a therapeutic surgical procedure to improve the symptoms of cardiovascular disease. Complications of CABG are physical and mental disorders that can lead to rehospitalization and post‐operative depression. Depression and coronary artery disease are highly comorbid conditions with estimates of comorbidity from 14% to 47% (Blumenthal et al., 2003). Studies have shown that post‐CABG depression can lead to mortality (Anda et al., 1993; Aromaa et al., 1994; Barefoot & MJC, 1996), decreased physical function (Rymaszewska et al., 2003), poor wound healing, and increased risk of cardiovascular events (angina and myocardial infarction) (Doering et al., 2005). The prevalence of depression after CABG is about 20% to 25% (McKhann et al., 1997; Pirraglia et al., 1999).

Depression can affect cardiovascular health, quality of life, and mortality after CABG (Borowicz Jr et al., 2002; Burg, Benedetto, Rosenberg, & Soufer, 2003; Burg, Benedetto, & Soufer, 2003; Doering et al., 2005; Oxlad et al., 2006). The prevalence of major depression among hospitalized patients due to CAD is 17%–27% (Lespérance et al., 2007). Patients with depressive symptoms before the first MI have been shown to have a higher risk for heart failure than non‐depressed patients (Dickens et al., 2006). Depression is a cardiac risk factor (Frasure‐Smith & Lespérance, 2005).

Hesperidin is a glycosylated flavonoid derived from the word hesperidium (fruit of the citrus family). The sugar‐free part is called hesperidin. Hesperidin is the most abundant flavonoids in oranges, mandarins, and lemons (Dolzhenko et al., 2010; Peterson et al., 2006). This flavonoid has antioxidant, anti‐inflammatory, anticancer, and antiallergic properties (Garg et al., 2001). The protective role of hesperidin in cardiovascular disease and depression is due to its anti‐inflammatory and antioxidant properties (Parhiz et al., 2015). The beneficial effects of hesperidin on the treatment of myocardial toxicity through activation of PPAR‐γ are reported (Agrawal et al., 2014).

P‐selectin acts as a cell adhesion molecule (CAM) on the surfaces of endothelial‐activated cells (McEver et al., 1989). In the atherosclerosis process, increasing the reactive oxygen species in the blood vessels upregulates the adhesion molecules (P‐selectin) on the vascular endothelium (Palmefors et al., 2014). Platelet activation plays an important role in the etiology of cardiovascular disease (Jin et al., 2007). Hesperidin can inhibit collagen and arachidonic acid‐induced platelet aggregation by inhibiting PLC‐γ2 phosphorylation and cyclooxygenase‐1. Serotonin also plays a role in platelet aggregation, and hesperidin has an antiplatelet role by inhibiting serotonin secretion (Jin et al., 2007).

LDL is an important and effective factor in the process of atherosclerosis. In endothelial cells of the arterial wall, macrophages attack LDL and cause LDL oxidation by stress oxidative (Palmefors et al., 2014) Oxidized LDL (OX‐LDL) has a significant effect on chronic inflammation that stimulates the release of chemotactic agents, cytokines, and growth factors from arterial wall cells (Witztum & Steinberg, 1991). Flavonoids are powerful antioxidants that can prevent LDL oxidation by increasing antioxidant capacity (Rice‐Evans et al., 1995, 1996; Van Acker et al., 1996). In the flavanone group, hesperidin and its glycoside hesperidin are more potent antioxidants.

According to evidence, it can be expected that hesperidin, as an antioxidant and an anti‐inflammatory agent, has beneficial effects on oxidized LDL and P‐selectin. To our knowledge, this double‐blind randomized clinical trial is the first study to investigate the effects of pure hesperidin supplementation on inflammatory markers (P‐selectin and hs‐CRP), oxidative factor (OX‐LDL), lipid profile, and blood pressure in post‐CABG patients with depressive symptoms, therefore we aimed to determine the effects of hesperidin supplementation on the serum levels of inflammatory and stress oxidative markers (P‐selectin, OX‐LDL, and hs‐CRP) and lipid profile in depressed CABG patients. We proposed higher level of oxidative stress in depressed patients may be as a synergetic factor.

## 2 MATERIALS AND METHODS

### 2.1 Ethical considerations

The study protocol was approved by the Research Ethics Committee of Iran University of Medical Sciences, Iran (IR.IUMS.REC.1395.9321323002), and registered in the Iranian Registry of Clinical Trials (IRCT ID: IRCT201704099472N12). Written informed consent was obtained from all participants prior to their inclusion in the study.

### 2.2 Patients

Totally, 80 patients with a history of isolated CABG within 1 to 3 months were enrolled into study. Participants had to meet the following inclusion criteria: Beck depression inventory score above 10, age range 30–70 years, and body mass index (BMI) between 25 and 40, Do not use any supplement including antioxidants and vitamins in the past 3 months before the study, do not consume more than one red‐orange or two other citruses per day, the absence of any sensitivity (digestive and dermal) to any antioxidant and vitamin supplement. The exclusion criteria were as follows: left ventricular ejection fraction EF ≤ 30, patients who modify or increase their medication such as antilipid drugs during the study period, valve surgery, heparin and warfarin consumption, uncontrolled chronic disease (such as hepatic or respiratory disorders, renal failure, malignancies, and diabetes), vegetarian diet, compliance below 80%, high intake of flavonoid‐rich drinks per day (including green tea, coffee, and citrus juice), smoking, and the appearance of allergy symptoms during the study.

### 2.3 Experimental design

This study was a placebo‐controlled, double‐blind, randomized clinical trial that was performed to evaluate the effects of 200 mg pure hesperidin supplementation on the serum levels of inflammatory and stress oxidative markers and lipid profile in post‐CABG patients. Eighty post‐CABG patients who met all criteria were divided into two groups by stratified block randomization (intervention: *n* = 40, control: *n* = 40). Patients in the intervention and placebo group consumed one capsule of 200 mg pure hesperidin or starch after lunch per day for 12 weeks, respectively. Finally, 68 patients completed the study. The sample size in this study was determined using P‐selectin (considering other variables) according to the study of Heidari et al., (2015). Patients' medications included aspirin, statins, beta‐blockers, inhibitors of angiotensin II, nitrates, and diuretics which was similar in the two groups. During the study, all participants were asked to continue their eating habits and do not take any vitamin /mineral supplements or flavonoid‐fortified drinks. Dietary intakes, anthropometric indices, blood pressure, and physical activity were evaluated at the beginning and end of the study. The IPAQ questionnaire was used to assess the physical activity of patients. This questionnaire evaluates participants' activity in five major domains (work, transportation, housework, sport, and leisure time physical activity).

### 2.4 Treatment

The hesperidin used in this study was produced by a Canadian Pharmaceutical Company (Kripps Pharmacy). Each capsule contained 200 mg of hesperidin extracted from the citrus aurantium. The selected dose was determined based on previous studies (Constans et al., 2015; Perche et al., 2014). Placebo supplements containing 200 mg of starch were made quite similar to the hesperidin supplements at the faculty of Pharmacy Tehran University of Medical Sciences.

### 2.5 Clinical assessments

At the beginning of the study, patients were asked to complete the Beck Depression Inventory (BDI) (Ghassemzadeh et al., 2005) to determine depressive symptoms. The weight of the patients was measured using fasting mode with minimum clothing and no shoes, and height in a standing position without shoes. Body mass index (BMI) is defined as weight (in kg) divided by the square of height (in meters).

Patients' physical activity was assessed by using International Physical Activity Questionnaires (IPAQ) (Wolin et al., 2008). Dietary intakes were evaluated by 24‐hour recall questionnaires for 3 days (2 days nonholiday and one holiday) at baseline and end of the study which was analyzed using Nutritionist IV software (First Databank), which has been modified for Iranian foods software.

### 2.6 Laboratory assessments

Whole blood venous sampling was done after 12 h of fasting. Blood samples were centrifuged to isolate serum. Sera samples were frozen at −80°C. Hs‐CRP was measured using immunoturbidometric method (CAT number: 516020; Pars Azmoon Co.). Plasma levels of P‐selectin (CAT number: E0201Hu; Shanghai Crystal Day Biotech Co., Ltd) and oxidized LDL (CAT number: E3225Hu; Shanghai Crystal Day Biotech Co., Ltd) were measured by enzyme‐linked immunosorbent assay (ELISA) method. Lipid profile including triglyceride (CAT number: 132500; Pars Azmoon Co.), HDL‐cholesterol (CAT number: 312050; Pars Azmoon Co.), LDL‐cholesterol (CAT number: 123050; Pars Azmoon Co.), total cholesterol (CAT number: 110500; Pars Azmoon Co.), and fasting blood sugar (CAT number: 117500; Pars Azmoon Co.) were measured using the enzymatic photometric method. All measurements were made according to the manufacturer's instructions.

### 2.7 Primary and secondary outcomes

Primary outcomes of the study were inflammatory and oxidative factors concentration in serum (hs‐CRP, P‐selectin, and oxidized LDL). Secondary outcomes were lipid profile, FBS, SBP, and DBP.

### 2.8 Statistical analyses

Statistical data were analyzed by Statistical Package for Social Sciences (SPSS) version 23. Normality of data distribution was assessed using Kolmogorov–Smirnov test. Paired sample *t*‐test was used to compare the means of quantitative variables (if their distribution was normal) in each group and independent sample *t*‐test was used to compare the means between the two groups. Wilcoxon test was used to compare them in each group and the Mann–Whitney test was used to compare them between two groups if their distribution was not normal.

Chi‐square and Fisher tests were used for qualitative variables. Covariance analysis was used to eliminate the effects of confounding factors that were significantly different between the two groups at baseline and during the study. *p* Values < .05 were considered significant.

## 3 RESULTS

**FIGURE 1:**
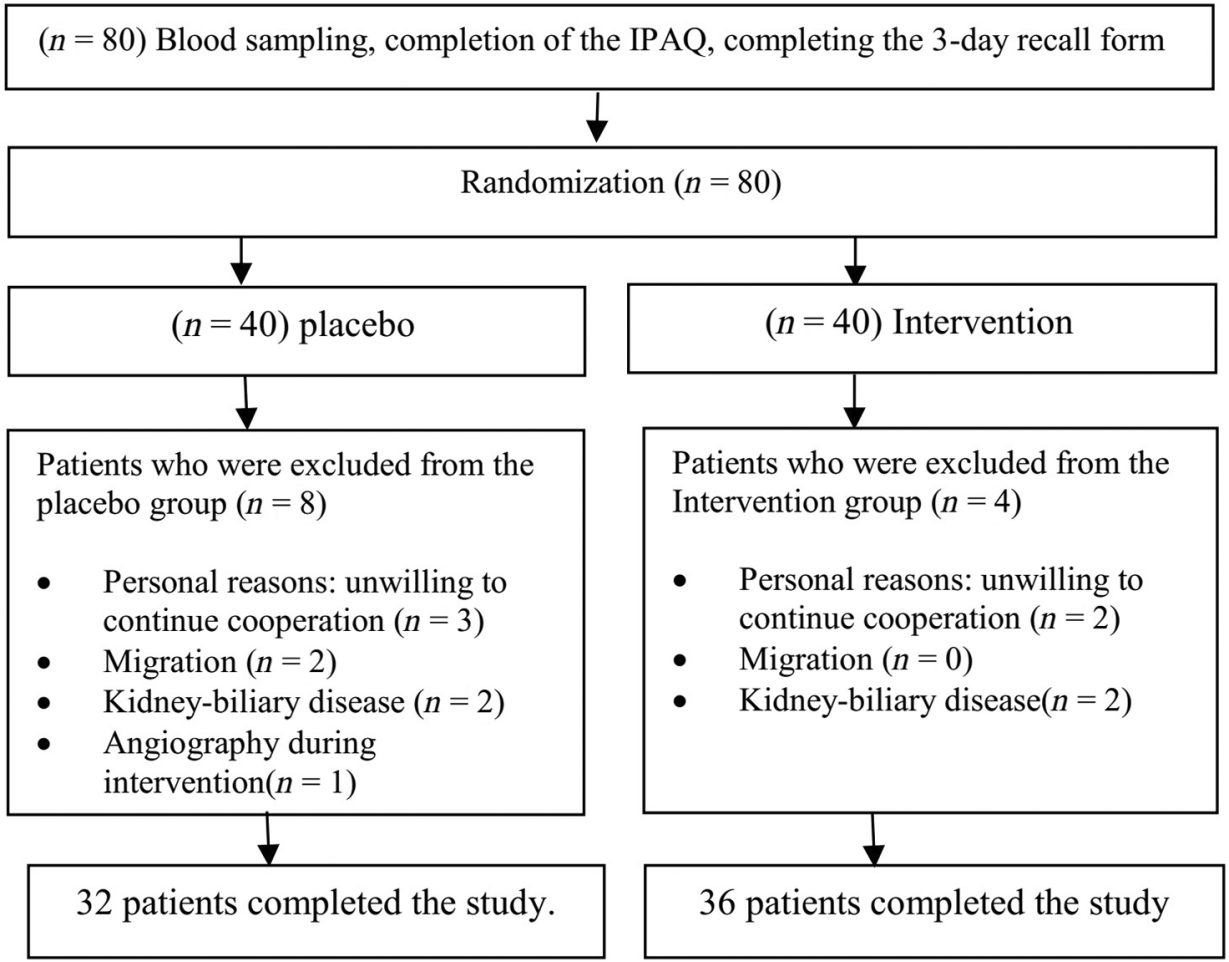
Flowchart of study participants.

From September 2018 to March 2019, 80 patients were recruited. Twelve patients discontinued the study (4 patients in intervention group and 8 patients in placebo group). Finally, 68 patients remained and completed the study (Figure 1). At the end of the study, participants reported no side effects.

The demographic and anthropometric characteristics of the participants at the beginning of the study are presented in Table 1. There were no significant differences in age and height between the two groups (*p* > .05) at baseline. There was no statistically significant difference in the ratio of males and females between the two groups (*p* > .05) too. Also, according to the results of this table, there was no significant difference between hesperidin and placebo groups in the duration of CABG surgery at the base. No significant changes were observed between the two groups at the base and at the end of the study in BMI and weight (*p* > .05). The physical activity did not differ at the baseline and end of the study between and within groups (*p* > .05) (Table 2).

**TABLE 1:** Baseline demographic and anthropometric characteristics of the study patients.

Characteristics	Hesperidin (*n* = 36)	Placebo (*n* = 32)	*p*‐Value
Demographic			
Age (y)^a^	59.75 ± 7.45	58.56 ± 7.16	.5^b^
Gender *n* (%)			
Male	25 (69.4)	25 (78.1)	.41^c^
Female	11 (31.6)	7 (21.9)	.41^c^
Duration of CABG (month)^d^	1.5 (0.5)	1.5 (0.5)	.13^c^
Anthropometric			
Height (cm)^a^	165.75 ± 8.39	168.03 ± 8.48	.27^b^
Weight (kg)^a^	76.59 ± 10.77	75.30 ± 6.49	.55^b^
BMI (kg/m^2^)^d^	27.25 (4.82)	25.90 (3.10)	.12^e^

*Note: p* Value < .05 has been considered significant.

Abbreviations: BMI, body mass index; CABG, coronary artery bypass surgery graft.

^a^ Normal values as mean ± SD.

^b^ Independent sample *t*‐test.

^c^ Chi‐square test.

^d^ Abnormal values as median (Q1−Q3).

^e^ Mann–Whitney Test.

**TABLE 2:** Physical activity of the study patients at the base and end of the treatment period.

Variable	Hesperidin		Placebo	*p*1^a^
IPAQ, Baseline, *n* (%)	Light	15 (41.7%)	13 (40.6%)	.93
	Moderate	21 (58.3%)	19 (59.4%)	
	Hard	0	0	
MET/min/week, End, *n* (%)	Light	16 (44.4%)	16 (50.0%)	.64
	Moderate	20 (55.6%)	16 (50.0%)	
	Hard	0	0	
	*p*2^b^	1	.25	

*Note: p* Value < .05 has been considered significant.

Abbreviations: IPAQ, International Physical Activity Questionnaire; p1, *p*‐value between groups; p2, *p*‐value within groups.

^a^ Chi‐square test.

^b^ McNemar test.

Dietary intakes of patients were evaluated by 3‐day diet recalls and records (2 days' nonholiday and one holiday) (Table 3). Based on dietary analyses, there were no significant changes between two groups at the baseline and end of the treatment period. However, significant differences were observed for EPA and iron intake within hesperidin group at the end of the study. The effect of these two variables was used as a confounder in covariance analysis.

**TABLE 3:** Dietary intakes of the study patients at the base and end of the treatment period.

Variables	Hesperidin (*n* = 36)	Placebo (*n* = 32)	*p*1
Energy (kcal)^a^			
Baseline	1578.45 ± 401.73	1544.89 ± 410.00	.75^b^
End	1606.51 ± 399.95	1651.06 ± 424.91	.66^b^
*p*2^c^	.40	.07	
Carbohydrate (g/d)^a^			
Baseline	233.71 ± 70.98	228.75 ± 69.54	.78^b^
End	244.67 ± 70.83	248.26 ± 67.99	.81^b^
*p*2^c^	.14	.06	
Protein (g/d)^a^			
Baseline	74.62 ± 19.87	73.44 ± 21.63	.77^b^
End	70.94 ± 20.24	75.09 ± 21.05	.38^b^
*p*2^c^	.19	.58	
Fat (g/d)^a^			
Baseline	40.36 ± 15.66	40.52 ± 13.78	.86^b^
End	40.00 ± 12.94	42.02 ± 16.67	.68^b^
*p*2^c^	.81	.20	
SFA (g/d)^a^			
Baseline	12.19 ± 4.65	11.74 ± 4.80	.67^b^
End	12.65 ± 5.42	12.01 ± 4.08	.88^b^
*p*2^c^	.67	.60	
MUFA (g/d)^a^			
Baseline	13.45 ± 6.54	13.35 ± 6.08	.97^b^
End	12.42 ± 4.98	14.21 ± 7.71	.37^b^
*p*2^c^	.48	.48	
PUFA (g/d)^d^			
Baseline	8.99(7.64)	8.40(4.57)	.67^e^
End	9.02(7.00)	9.86(9.24)	.88^e^
*p*2^f^	.87	.32	
Cholesterol (mg/d)^a^			
Baseline	211.93 ± 100.90	247.07 ± 155.18	.66^b^
End	216.87 ± 124.26	198.07 ± 90.01	.59^b^
*p*2^c^	.59	.47	
EPA (g/d)^d^			
Baseline	0.007(0.105)	0.008(0.073)	.55^e^
End	0.003(0.010)	0.006(0.080)	.18^e^
*p*2^f^	.009	.92	
Iron (mg/d)^a^			
Baseline	11.4 ± 4.06	11.54 ± 4.27	.86^b^
End	12.6 ± 6.03	12.7 ± 5.95	.98^b^
*p*2^c^	.04	.26	

*Note*: *p* Value < .05 has been considered significant.

Abbreviations: *p*1, *p*‐value between groups; *p*2, *p*‐value within groups.

^a^ Normal values as mean ± SD.

^b^ Independent sample *t*‐test.

^c^ Paired *t*‐test.

^d^ Abnormal values as median (Q1−Q3).

^e^ Mann–Whitney Test.

^f^ Wilcoxon test.

Table 4 shows the effects of hesperidin supplementation on serum levels of inflammatory and oxidative factors, fasting blood glucose, and lipid profile in post‐CABG surgery patients. Changes in hs‐CRP, OX‐LDL, and P‐selectin (within and between the intervention and placebo groups) were not statistically significant during the study (*p* > .05). Differences in inflammatory and oxidative variables at the end of the study remained statistically not significant after adjusting for age, BMI base values, and differences in BMI.

**TABLE 4:** Effects of hesperidin supplementation on inflammatory and oxidative markers, fasting blood sugar, and lipid profile.

Variables	Hesperidin (*n* = 36)	Placebo (*n* = 32)	*p*1	*p*2
hs‐CRP (mg/L)^a^				
Baseline	1.29 (1.64)	1.10 (2.63)	.354^b^	–
End	0.51 (3.7)	0.82 (3.75)	.253^b^	.389
*p*3^c^	.390	.708	–	–
P‐selectin (ng/mL)^a^				
Baseline	1.05 (1.15)	1.00 (0.52)	.468^b^	–
End	1.21 (0.59)	1.17 (0.40)	.782^b^	.371
*p*3^c^	.471	.059	–	–
OX‐LDL (ng/mL)^a^				
Baseline	276.15(278.85)	287.65 (219.7)	.572^b^	–
End	330.79(247.78)	294.3 (1445.43)	.302^b^	.549
*p*3^c^	.741	.172	–	–
FBS (mg/dL)^a^				
Baseline	115.5 (41.25)	103.00 (30.00)	.109^b^	–
End	111.5 (43.5)	104.50 (32.00)	.654^b^	.653
*p*3^c^	.643	.885	–	–
TG (mg/dL)^d^				
Baseline	120.08 ± 33.91	129.4 ± 26.93	.218^e^	–
End	116.72 ± 3.8	140.68 ± 28.51	.002^e^	.004
*p*3^f^	.40	.001	–	–
HDL‐C (mg/dL)^d^				
Baseline	38.07 ± 7.44	36.85 ± 6.69	.483^e^	–
End	43.38 ± 9.94	39.57 ± 7.62	.084^e^	.079
*p*3^f^	.003	.021	–	–
LDL‐C (mg/dL)^d^				
Baseline	61.76 ± 14.33	66.02 ± 14.73	.231^e^	–
End	66.02 ± 14.73	68.04 ± 16.31	.207^e^	.102
*p*3^f^	.32	.342	–	
TC (mg/dL)^d^				
Baseline	122.86 ± 31.86	127.18 ± 26.2	.546^e^	–
End	127.86 ± 34.55	131.96 ± 25.25	.564^e^	.407
*p*3^f^	.152	.199	–	

*Note*: *p* Value < .05 has been considered significant.

Abbreviations: FBS, fasting blood sugar; HDL‐C, high‐density lipoprotein cholesterol; LDL‐C, low‐density lipoprotein cholesterol; OX‐LDL, Oxidized LDL; *p*1, *p*‐value between groups; *p*2, results from analysis of covariance in the adjusted models (adjusted for age, BMI base values, diff BMI), *p*3, *p*‐value within groups; TC, Total cholesterol; TG, Triglyceride.

^a^ Abnormal values as median (Q1−Q3).

^b^ Mann–Whitney Test.

^c^ Wilcoxon test.

^d^ Normal values as mean ± SD.

^e^ Independent sample *t*‐tests.

^f^ Paired *t*‐test.

Triglyceride reduction between the two groups was significant and remained statistically significant after adjusting by covariance analysis (*p* = .004). The increase in HDL‐c was not significant (*p* > .05). Changes in fasting blood sugar, cholesterol, and LDL were not statistically significant (*p* > .05). Changes in these variables after adjusting for age, BMI base values, and differences in BMI also were not significant.

The changes in systolic and diastolic blood pressure during the study are shown (Table 5). SBP in the hesperidin group tended to decrease significantly (*p* = .09). DBP in the placebo group increased significantly (*p* = .001) but changes in systolic and diastolic blood pressure were not statistically significant between groups (*p* > .05).

**TABLE 5:** Effects of hesperidin supplementation on blood pressure in post‐CABG patients.

Variable	Hesperidin (*n* = 36)	Placebo (*n* = 32)	*p*1^a^
SBP (mmHg)^b^			
Baseline	126.5(22.75)	122.0(23.25)	.108
End	124.5(12.25)	120.0(14.25)	.406
*p*2^c^	.098	.893	
DBP (mmHg)^b^			
Baseline	78.5(13.75)	74.5(10.75)	.075
End	80.0(11.0)	79.0(8.00)	.826
*p*2^c^	.635	.001	

*Note*: *p* Value < .05 has been considered significant.

Abbreviations: DBP, diastolic blood pressure; *p*1, *p*‐value between groups; *p*2, *p*‐value within groups; SBP, systolic blood pressure.

^a^ Mann–Whitney Test.

^b^ Abnormal values as median (Q1−Q3).

^c^ Wilcoxon test.

## 4 DISCUSSION

To our knowledge, this double‐blind randomized clinical trial is the first study to investigate the effects of hesperidin on inflammatory markers (P‐selectin and hs‐CRP), oxidative factor (OX‐LDL), lipid profile, and blood pressure in post‐coronary artery bypass graft surgery patients with depressive symptoms. It was found that 200 mg of hesperidin supplementation for 12 weeks had no effect on hs‐CRP, P‐selectin, or oxidized LDL concentration. Studies investigating inflammatory and oxidative markers in humans are limited, especially in depressed post‐CABG patients.

In vitro study conducted by Liu et al. (2007) showed the role of 5 mmol/L hesperidin on LDL oxidation induced by Cu^2+^. In this study, it was observed that hesperidin could inhibit LDL oxidation due to its antioxidant properties. The results of our study were in line with Rangel‐Huerta et al. (2015), who showed that consumption of normal polyphenol juice contains 299 mg/d hesperidin and high polyphenol juice contains 745 mg/d hesperidin for 12 weeks in obese and overweight subjects in cross‐over study had no effect on OX‐LDL concentration.

In the current study, the changes in level of serum P‐selectin level were not significantly different in the hesperidin group compared to the placebo group. Our results concur with Salden et al., who showed that consumption of 450 mg/dl hesperidin in 68 overweight subjects for 6 weeks could not have a significant effect at the end of the study on P‐selectin and E‐selectin (Bouke et al., 2016). Contrary to this result, one in vitro study showed that hesperetin inhibited platelet aggregation induced by collagen and arachidonic acid via suppression of cyclooxygenase1 activity (Jin et al., 2007).

In a study conducted by Ferreira et al. (2016), the effects of 100 mg/kg of hesperidin on 60 rats with systemic inflammation and oxidative stress for 4 weeks reduced the hs‐CRP levels in the rats significantly. Haidari et al. (2015) observed that 600 mg of hesperidin supplementation in 75 patients with MI for 4 weeks did not decrease hs‐CRP significantly. Morand et al. (2010) in a study reported that 292 mg of hesperidin in a cross‐over study on 24 overweight healthy subjects for 4 weeks did not have a significant effect on hs‐CRP. The results of these two human studies are in line with ours.

Due to the high levels of inflammation and oxidative stress in CABG patients and the exacerbating effect of depression on the levels of these factors, we investigated the effect of hesperidin on depressed CABG patients, but studies on the anti‐inflammatory and oxidative effects of hesperidin are limited, especially in humans, and further studies are needed to investigate the effect of hesperidin on OX‐LDL and P‐selectin in humans. In vitro and animal studies reported that hesperidin reduced OX‐LDL, P‐selectin, and hs‐CRP, but most of the human studies did not show significant effects. It is possible that the dose of hesperidin is higher and the time of intervention is longer in animal studies than in human studies (Vabeiryureilai et al., 2015). In a study by Yamada et al. (2006), they found that glucosyl hesperidin (hesperetin) had more efficient absorption than hesperidin; therefore, in comparison to the same doses of hesperidin, it has higher bioavailability. As a result, the use of glucosyl hesperidin in animal studies could be an important reason for the beneficial effects of hesperidin on inflammatory factors in animal models (Lorzadeh et al., 2019). In our study, the dose of hesperidin was insufficient to effect inflammatory and oxidative markers.

Studies have shown that high levels of HDL cholesterol and triglycerides are associated with depression. Therefore, the effect of hesperidin on CABG patients with depression was investigated. Our study showed that hesperidin significantly reduced triglyceride. The improvement in HDL was also observed at the end of the intervention compared to the baseline values within groups, but the difference between the hesperidin and placebo groups was not statistically significant (but nearly significant).

Animal studies have shown that hesperidin may decrease serum TG by suppressing pancreatic lipase (Kawaguchi et al., 1997). Consistent with our study results, Miwa et al. in a clinical trial study on 40 hypercholesterolemic subjects for 6 weeks have reported that G‐hesperidin has a favorable effect on triglyceride, but not on serum cholesterol. According to their study, mechanisms of hesperidin in reduction for triglycerides are related to LPL activation and facilitation of removing TG from TRL (triglyceride‐rich lipoprotein). These effects of hesperidin may inhibit the formation of small dense LDL, which may have a role in preventing atherosclerosis progression (Miwa et al., 2004).

Haidari et al. (2015) showed that 600 mg of hesperidin supplementation for 4 weeks increased HDL level in patients with myocardial infarction. In another study, Kurowska et al. (2000) reported that consumption of 750 mL orange juice per day for 4 weeks could increase HDL‐c levels in hypercholesterolemic subjects. Increased serum HDL levels could be related to the antioxidant properties of hesperidin, which increase PON1, and in previous studies, the relationship between paraoxonase activity with HDL‐c and their role in atherosclerosis has been confirmed (Haidari et al., 2012). The significant increase in serum HDL‐c in the intervention and placebo groups in our study could be due to statin consumption and lifestyle changes in patients (Haidari et al., 2015), but the increase in the Hesperidin group was greater than the placebo (near significant). According to our observation that TG has decreased significantly after the study and borderline enhancement of HDL‐c in the intervention group, this result may be related to the role of hesperidin in increasing VLDL catabolism and its effect on TG removal by CETP (cholesterol ester transfer protein) (Miwa et al., 2005).

In our study, cholesterol and LDL changes in both groups and between the two groups at the beginning and the end of the study were not statistically significant. Rizza et al. (2011) demonstrated that 3 weeks of supplementation with 500 mg/d of hesperidin decreases total cholesterol levels in 24 subjects with metabolic syndrome. Based on previous studies, cholesterol‐lowering effects in this study were attributed to the role of hesperidin in inhibiting APO B100 secretion from hepatocytes (Rizza et al., 2011; Wang et al., 2011). Animal studies have suggested that the effects of hypocholesterolemic hesperidin are related to decreased hepatic HMG‐COA reductase and cholesterol acyltransferase activity and increased fecal cholesterol (Jung et al., 2006). The results are different from our study results but this may be related to the body's compensatory and hemostatic response to cholesterol‐lowering effect of hesperidin (Demonty et al., 2010) and it seems that prolonged intervention is needed for the effect of hesperidin on HMG‐COA reductase because its sensitivity to hesperidin in humans is lower than in rats (Miwa et al., 2005).

In the current study, it was shown that hesperidin tended to decrease SBP significantly in the intervention group. But at the end of the study, systolic and diastolic differences between the two groups were not significant. Wang and colleagues observed that the administration of hesperidin in rats fed a high‐cholesterol diet did not reduce blood pressure significantly (Wang et al., 2011). Haidari et al. (2015) reported that 600 mg hesperidin supplementation in patients with myocardial infarction did not change blood pressure during intervention. The results of these two studies were in line with our study results. Morand et al. (2010) demonstrated that consumption of orange juice (292 mg hesperidin) in healthy overweight individuals for 4 weeks decreased DBP significantly. Previous animal studies have shown the antihypertensive effects of hesperidin through increased endothelial nitric oxide synthase (eNOS) activity and NO production (Chiou et al., 2008). Another mechanism of hesperidin lowering blood pressure could be through angiotensin‐converting enzyme inhibition (Actis‐Goretta et al., 2006).

The limitations of our study are the unequal number of sample sizes in the two intervention and placebo groups no evaluation of the bioavailability of hesperidin, no measurement of hesperidin concentration in serum. It was also useful to evaluate the antioxidant role of hesperidin on LDL oxidation by measuring other indices such as PON‐1 and TBARS. Further studies are recommended to investigate the best and most effective dose of hesperidin in humans and evaluate the mechanism of the effects of hesperidin in humans.

In conclusion, this randomized double‐blind placebo‐controlled trial showed that consumption of 200 mg hesperidin could decrease serum levels of triglyceride in the hesperidin group compared to the placebo at the end of the intervention significantly. This effect of hesperidin can be effective in preventing the progression of atherosclerosis in CVD patients after CABG surgery. Significant improvement in HDL levels was observed at the end of the study compared to the baseline values in groups, but changes between two groups were not statistically significant (but nearly significant). However, hesperidin could not show a significant effect on other lipid parameters and inflammatory and oxidative factors in post‐CABG patients. Hesperidin tended to reduce SBP in the intervention group, but at the end of the study, systolic and diastolic differences between the two groups were not significant.

## AUTHOR CONTRIBUTIONS

**Zahra Booyani:** Data curation (equal); writing – original draft (equal). **Naheed Aryaeian:** Conceptualization (equal); project administration (equal); supervision (equal). **Negar Omidi:** Conceptualization (equal). **Atie Khorasanian:** Data curation (equal); writing – original draft (equal). **Agha Fatemeh Hoseini:** Formal analysis (equal). **Mostafa Nejatian:** Conceptualization (equal). **Shima Jazayeri:** Conceptualization (equal). **Mehrnaz Morvaridi:** Writing – original draft (equal); writing – review and editing (equal).

## FUNDING INFORMATION

The Iran University of Medical Sciences financially supported this clinical trial by grant ID: 95‐04‐27‐29848.

## CONFLICT OF INTEREST STATEMENT

The authors have declared that there is no conflict of interest.

## Data Availability

The data that support the findings of this study are available from the corresponding author upon reasonable request.
